# Role of treatment for solitary pulmonary nodule in breast cancer patients

**DOI:** 10.1186/1477-7819-9-124

**Published:** 2011-10-11

**Authors:** Masahiro Kitada, Kazuhiro Sato, Yoshinari Matsuda, Satoshi Hayashi, Naoyuki Miyokawa, Tadahiro Sasajima

**Affiliations:** 1 Department of Surgery, Asahikawa Medical University, Midorigaoka-Higashi 2-1-1-1 Asahikawa Hokkaido 078-8510, Japan; 2 Department of Clinical Pathology, Asahikawa Medical University, Midorigaoka-Higashi 2-1-1-1 Asahikawa Hokkaido 078-8510, Japan

## Abstract

**Background:**

Metastatic pulmonary tumors secondary to breast cancer detected either before or after surgery are predominantly multiple and bilateral. However, in cases detected to have a solitary pulmonary nodule (SPN), determining whether the lesion represents a primary cancer, metastasis, or a benign pulmonary lesion can be difficult.

**Materials and methods:**

Between January 2000 and December 2009, we performed breast cancer surgery on 1,226 patients, of which 49 cases (3.9%) were detected to have pulmonary lesions before or after the surgery. In 14 of these patients, video-assisted thoracoscopic surgery was performed to remove a SPN.

**Result:**

Pathological examination of the resected specimens in these 14 cases revealed metastatic pulmonary tumor in 8 cases, primary lung cancer in 3 cases, and benign disease in 3 cases. While lobectomy was performed in one of these patients with metastatic pulmonary tumor, the remaining 7 underwent partial resection of the lung. The primary lung cancer was an adenocarcinoma in all 3 patients, and lobectomy plus mediastinal lymph node dissection was performed in these patients. The tumor grading based on pathological diagnosis was T1N0M0, p-Stage 1A in all 3 patients. The prognosis was good in the breast cancer patients in whom the metastatic lung tumor was a SPN.

**Conclusion:**

Evaluating the immunohistochemical cytokeratin profile and levels of the TTF-1 and GCDFP-15 of the lesion was useful when distinguishing between pulmonary cancer and metastatic pulmonary tumor. In addition, some patients exhibited changes in the biological properties of the metastatic tumor, and delete tumor resection by video-assisted thoracoscopic surgery can be useful for deciding the drug treatment strategy in some cases

## Background

Metastasis from breast cancer predominantly involves the bones, lungs, and liver. Local therapy of metastases resulting in radical cure is extremely rare, and systemic pharmacotherapy is usually selected as the basic therapeutic approach, with the aim of prolonging life, alleviating symptoms, and improving the quality of life (QOL) in breast cancer patients with metastatic tumors. Patients with metastatic pulmonary lesions often have multiple lesions and/or pleural effusion. Definitive diagnosis is achieved by imaging modalities, and pharmacotherapy is administered. However, in the case of a solitary pulmonary nodule (SPN), distinguishing between primary lung cancer, metastatic pulmonary tumor, and a benign pulmonary lesion can be difficult. In addition, in the case of metastatic pulmonary tumors, differences in the biological properties of the tumor (e.g., estrogen receptor (ER), progesterone receptor (PgR) and human epidermal growth factor receptor type 2 (HER2) status, relative to those of the primary tumor may require revisions of the therapeutic strategy. We reviewed our experience of such patients treated by us at our institution.

## Materials and methods

From January 2000 through December 2009, we performed breast cancer surgery on 1,226 patients, and identified pulmonary lesions in 49 of these patients (3.9%), either before or after the surgery. In 35 of these patients, we made the diagnosis of metastatic breast cancer on the basis of detection of multiple lesions and/or pleuritis carcinomatosa, and pharmacotherapy was initiated. The present review focuses on the remaining 14 patients who underwent thoracoscopic surgery for removal of a SPN of indeterminate diagnosis that was located in the peripheral lung. All the 14 patients were women, with a mean age of 58.4 years (range, 44-76 years). Three women were premenopausal, and the remaining 11 were postmenopausal.

In our breast cancer patients who were asymptomatic, we conducted periodic checkups for the detection of pulmonary lesions, including chest computed tomography (CT), before breast cancer surgery and then annually for 10 years following the surgery. When a SPN of indeterminate diagnosis was detected, resection was performed by video-assisted thoracoscopic surgery (VATS) if the lesion was a solid mass measuring ≥ 1.0 cm in diameter. In the case of a nodular lesion or ground-glass opacity (GGO) measuring < 1.0 cm in diameter, VATS was performed after 3-6 months, if re-examination showed no tendency towards shrinkage of the tumor. The resected specimens were subjected to intraoperative rapid pathological diagnosis, and the subsequent operative method was determined based on the findings. At the case to have thought that the identification of the nodule was difficult about, it did a marking under the CT guide before the operation. We detained a hook wire with the thread in the nodule. In the case of lesions for which distinguishing between primary lung cancer and metastatic breast cancer during surgery was difficult, the therapeutic strategy was determined based on the final diagnosis reached by final histopathological examination of the resected specimens.

## Results

### 1. Histological diagnoses of SPNs

Histopathological examination of the resected SPNs in the 14 patients showed metastatic pulmonary tumor in 8 patients (57.1%), primary lung cancer in 3 patients (22.0%), and benign disease in the remaining 3 patients. While lobectomy was performed in one of these patients with metastatic pulmonary tumor, the remaining 7 underwent partial resection of the lung. The primary lung cancer was an adenocarcinoma in all 3 patients, and lobectomy +mediastinal lymph node dissection was performed in these patients. We enforced immediately lobectomy and lymohadenectomy in 2 cases which diagnosed lung cancer during enforcement of thoracoscopy. 1 case which was not diagnosed lung cancer enforced lobectomy after some time. The tumor grading based on pathological diagnosis was T1N0M0, p-Stage 1A in all 3 patients. The benign disease was mycobacteriosis in 2 patients and mycosis in 1 patient. Partial pulmonary resection were performed in all 3 cases.

### 2. Tumor size

Tumor diameter in the 8 patients with metastatic pulmonary tumors was < 1.0 cm in 4 patients, 1.0 to 2.0 cm in 3 patients, and > 2.0 cm in 1 patient. The diameter of the primary lung cancer tumor was < 1.0 cm in 1 patient and 1.0 to 2.0 cm in the remaining 2 patients, the difference not being significant. In addition, the lesion diameter in the case of benign lung disease was 1.0 to 2.0 cm in 2 patients and > 2.0 cm in 1 patient.

### 3. Outcomes of the patients with metastatic breast cancer (Tables 1, 2)

**Table 1:** Cases of lung metastasis from breast cancer

Case	Age	p-TNM	Pathology	Grade	DFI
1	55	T1N0M0	Scirrhous ca.	II	9 M

2	68	T1N0M0	Mucinous ca.	II	63 M

3	66	T1N0M0	Scirrous ca.	II	48 M

4	69	T2N1M0	Invasive micropapillary ca.	I	13 M

5	59	T2N0M0	Scirrhous ca.	II	37 M

6	61	T2N0M0	Scirrhous ca.	II	26 M

7	65	T2N1M0	Solid-tubular ca.	II	12 M

8	44	T1N1M0	Scirrhous ca.	II	34 M

DFI: disease-free interval; TNM, TNM classification; Grade, nuclear grade; ca., carcinoma.

**Table 2:** Changes in biochemical characteristics lung metastasis from breast cancer

Case	ER	PgR	HER2	ER*	PgR*	HER2*	Therapy	Result
1	-	-	-	-	-	-	-	76A

2	-	-	1+	-	-	1+	-	48A

3	1+	-	-	-	-	-	Chemo	35A

4	2+	1+	-	2+	1+	-	Chemo	35D

5	2+	3+	3+	2+	1+	3+	Endocrine	34A

6	2+	-	3+	-	-	3+	Chemo+Tr	26A

7	-	-	3+	-	-	3+	Chemo+Tr	25A

8	1+	3+	3+	2+	2+	2+	Endocrine+Tr	18A

ER, estrogen receptor; PgR, progesterone receptor; HER2, human epidermal growth factor receptor type 2; ER*, ER after resection; PgR*, PgR after resection; HER2*, HER2 after resection; Chemo, chemotherapy; Endocrine, endocrine therapy; Tr, trastuzumab; A, alive; D,death

One of the 8 patients with metastatic pulmonary tumor showed metastases to multiple organs and died within 15 months after the breast surgery, whereas the remaining other 7 patients were still alive as of this writing. On the other hand, the MST (median survival time) in the patients with pulmonary metastases, including those with changed biochemical-characteristics of the metastatic lung tumor (8 cases), those with multiple-metastases, and those with metastases to other organs, was 37 months. The prognosis was good in the breast cancer patients in whom the metastatic lung tumor was a SPN. The biological properties of the metastatic lesion differed from those of the primary lesion in 2 patients: ER(1+), PgR(-), HER2(-) changing to ER(-), PgR(-), HER2(-) in 1 patient, and ER(2+), PgR(-), HER2(3+) changing to ER(-), PgR(-), HER2(3+) in the other patient. Treatments for those 2 patients consisted of weekly paclitaxel (PTX) for 1 patient and trastuzumab plus weekly PTX for the other.

### 4. Case report

**Figure 1:**
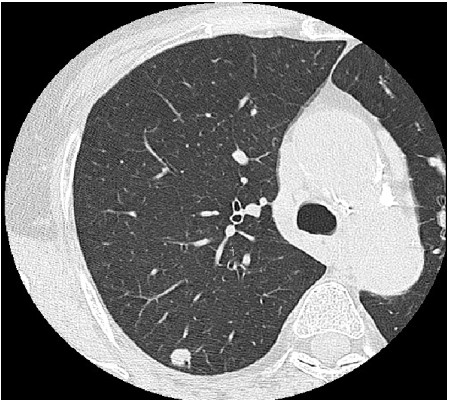
**CT image**. A nodular shadow, 10 mm in diameter, is seen in the right S10 region.

**Figure 2:**
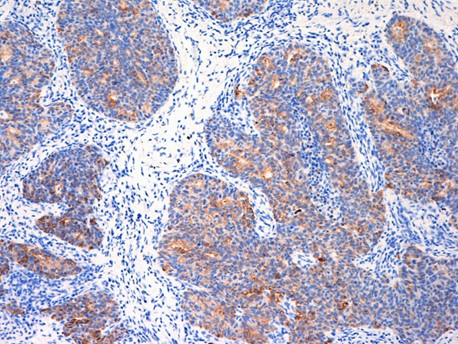
**CK-7-positive tumor**. CK-7-positive tumor of immunohistochemical staining (×100) cells.

**Figure 3:**
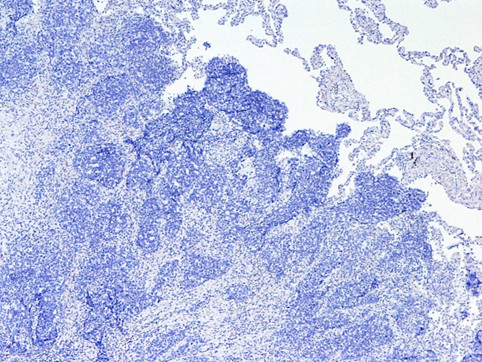
**CK-20-negative tumor cells**. CK-20-negative tumor cells of immunohistochemical staining (×100) cells.

**Figure 4:**
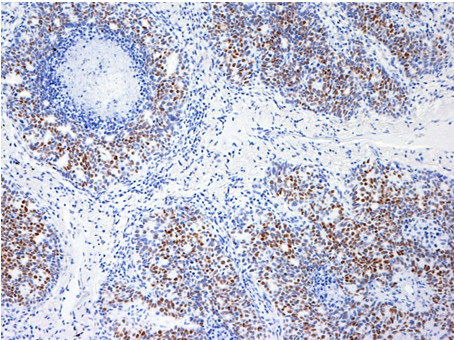
**TTF-1-positive tumor cells**. TTF-1-positive tumor cells of immunohistochemical staining (×100) cells.

**Figure 5:**
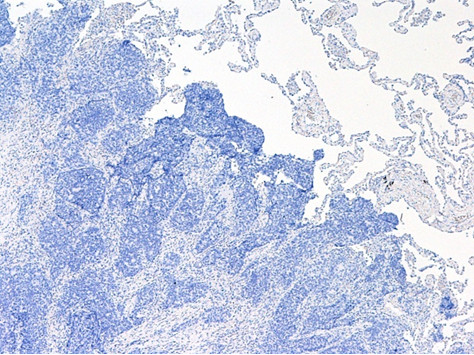
**GCDFP-5-negative tumor cells**. GCDFP-5-negative tumor cells of immunohistochemical staining (×100) cells.

A 65-year-old Japanese woman had undergone standard mastectomy 6 years earlier, and had been administered postoperative chemotherapy on the basis of the biological properties of the drug, namely, t2n1, ER(-), and PgR(-). While the clinical course was being monitored, periodic, thoracic CT examination revealed a pulmonary mass shadow measuring 1.2 cm in diameter in the right S10 (Figure 1). This lesion had not been seen on the CT performed in the previous year. The possibility of primary lung cancer could not be ruled out based only on the CT finding, therefore, VATS resection was performed. It was not possible to determine by intraoperative rapid pathological diagnosis whether the lesion represented a primary lung cancer or a metastatic tumor, and the surgery was completed. Immunohistochemical staining of the resected specimen showed a cytokeratin (CK) profile of CK7(+) and CK20(-), characteristic of pulmonary adenocarcinoma, while the tumor tissue was also positive for thyroid transcription factor (TTF)-1, a lung cancer marker, and negative for gross cystic disease fluid protein (GCDFP)-15, a breast cancer marker. The tumor was thus diagnosed as a primary lung cancer (Figure 2, 3, 4 and 5). Three weeks later, right upper lobectomy and mediastinal lymph node resection were performed. The final diagnosis in the tumor was moderately differentiated adenocarcinoma, p-T1N0M0. No adjuvant chemotherapy was administered, and the patient remains alive with no evidence of recurrence, as of this writing.

## Discussion

Pulmonary metastases following surgery for breast cancer are usually detected as multiple pulmonary nodules and/or pleural carcinomatosis, representing multiple nodules and/or pleural effusion or lymphatic carcinomatosis caused by malignant pleuritis. Diagnosis is normally comparatively easy. However, in the case of detection of a SPN on preoperative examination or during postoperative follow-up of breast cancer patients, accurate diagnosis can be very difficult. Mery et al. [1] performed histological diagnosis of SPNs in approximately 300 patients with antecedent cancers. They reported that approximately 80% of the patients had malignant disease, with primary pulmonary carcinoma in 41% and metastatic pulmonary tumors in 38% of the patients. Quint et al. [2] studied 161 patients and found that 50.3% had primary pulmonary carcinoma and 31.1% had metastatic pulmonary tumor. In addition, Rena et al. [3] reported that, among 79 patients who had undergone radical surgery for breast cancer, 48.1% developed primary pulmonary carcinoma and 30.4% developed metastatic pulmonary tumors. The corresponding incidences at our institution were 22.1% and 57.1%, respectively. Our combined incidences of approximately 80% for primary and metastatic malignant pulmonary tumors is consistent with the incidences reported by others, however, the percentage of metastatic pulmonary tumors in our series was higher. This can be attributed to the fact that our base analytical population comprised patients who had undergone radical surgery for breast cancer at our institution and did not include patients who had been referred to us after radical surgery performed at another institution. In any case, in the event of detection of a SPN, the possibility of primary pulmonary carcinoma should be borne in mind.

The size of the pulmonary tumor was ≤ 2.0 cm in all but 2 of our patients. This can be ascribed to the fact that the periodic CT examinations facilitated early detection of tumors in almost all patients. Burden et al. reported that 41.7% of tumors measuring < 1.0 cm in diameter were malignant, demonstrating that malignancy is not unusual even in tumors with such a small diameter [4]. Recent years have seen increased application of VATS, allowing less invasive pulmonary resection as compared to conventional open-chest surgery. VATS is now performed at many institutions, and its usefulness has been documented [5,6].

We perform intraoperative rapid pathological diagnosis, but differentiating between pulmonary adenocarcinoma and metastatic pulmonary tumor can be difficult. Examination of permanent specimens is therefore sometimes necessary for a definitive diagnosis, on immunohistochemical staining, pulmonary and thyroid adenocarcinomas characteristically shows that TTF-1 [7]. In addition, staining for the glycoprotein GCDFP-15, a marker of breast cancer, and for CK7 and CK20 is also considered to be useful for making a definitive diagnosis [8,9]. In the case reported above, we were unable to reach a definitive diagnosis during surgery. However, we obtained a definitive diagnosis of pulmonary adenocarcinoma with an immunostaining profile of CK7(+), CK20(-), GCDFP-15(-) and TTF-1(+) by examination of permanent resected specimens.

Another important point that must be taken into consideration in the case of metastatic pulmonary tumors in breast cancer patients is the possibility of changes in the original molecular biological profile of the tumor. In two of our patients, the primary breast cancers was ER-positive, whereas the metastatic tumor was negative for this marker. This phenomenon is sometimes seen even in patients with local recurrence or recurrence in other organs. With regard to treatments administered following lung surgery, tailoring treatment to the biological characteristics of the pulmonary metastatic tumor is imperative. We administered anticancer drugs as postoperative treatment. Based on our present experience, when pharmacotherapy exerts inadequate efficacy in a patient with multiple lung metastases from breast cancer, the possibility of changes in the molecular biological properties of the metastatic tumor should be considered. If the patient is in good overall health, consideration should be given to performing tumor biopsy via VATS.

## Conclusions

In the case of detection of a SPN, determining whether the lesion represents primary cancer, metastasis or a benign pulmonary lesion can be difficult. Evaluating the immunohistochemical cytokeratin profile of the lesion in terms of CK7 and CK20 was useful when distinguishing between pulmonary adenocarcinoma and metastatic pulmonary tumor was difficult, along with levels of the lung cancer marker TTF-1 and the breast cancer marker GCDFP-15. In addition, some patients exhibited changes in the biological properties of the metastatic tumor, and delete tumor resection by video-assisted thoracoscopic surgery can be useful for deciding the drug treatment strategy in some cases

## Competing interests

The authors declare that they have no competing interests.

## Authors' contributions

MK have operated this case and analyzed all data. KS, and YM, SH did the assistant of the operation. NM diagnosed h the pathology of this case.

TS was the professor of the surgical science and had a guide.

All authors read and approved the final manuscript.
